# Saliva DNA quality and genotyping efficiency in a predominantly elderly population

**DOI:** 10.1186/s12920-016-0172-y

**Published:** 2016-04-07

**Authors:** Harini V. Gudiseva, Mark Hansen, Linda Gutierrez, David W. Collins, Jie He, Lana D. Verkuil, Ian D. Danford, Anna Sagaser, Anita S. Bowman, Rebecca Salowe, Prithvi S. Sankar, Eydie Miller-Ellis, Amanda Lehman, Joan M. O’Brien

**Affiliations:** Scheie Eye Institute, University of Pennsylvania, 51 N. 39th Street, Philadelphia, PA 19104 USA; Illumina Inc, San Diego, CA USA

**Keywords:** Saliva, Blood, DNA, Genotyping, Microarrays, GWAS, SNPs, Elderly, Glaucoma, African-Americans, 260/280 absorbance, Call rates

## Abstract

**Background:**

The question of whether DNA obtained from saliva is an acceptable alternative to DNA from blood is a topic of considerable interest for large genetics studies. We compared the yields, quality and performance of DNAs from saliva and blood from a mostly elderly study population.

**Methods:**

Two thousand nine hundred ten DNAs from primarily elderly subjects (mean age ± standard deviation (SD): 65 ± 12 years), collected for the Primary Open-Angle African-American Glaucoma Genetics (POAAGG) study, were evaluated by fluorometry and/or spectroscopy. These included 566 DNAs from blood and 2344 from saliva. Subsets of these were evaluated by Sanger sequencing (*n* = 1555), and by microarray SNP genotyping (*n* = 94) on an Illumina OmniExpress bead chip platform.

**Results:**

The mean age of subjects was 65, and 68 % were female in both the blood and saliva groups. The mean ± SD of DNA yield per ml of requested specimen was significantly higher for saliva (17.6 ± 17.8 μg/ml) than blood (13.2 ± 8.5 μg/ml), but the mean ± SD of total DNA yield obtained per saliva specimen (35 ± 36 μg from 2 ml maximum specimen volume) was approximately three-fold lower than from blood (106 ± 68 μg from 8 ml maximum specimen volume). The average genotyping call rates were >99 % for 43 of 44 saliva DNAs and >99 % for 50 of 50 for blood DNAs. For 22 of 23 paired blood and saliva samples from the same individuals, the average genotyping concordance rate was 99.996 %. High quality PCR Sanger sequencing was obtained from ≥ 98 % of blood (*n* = 297) and saliva (*n* = 1258) DNAs. DNA concentrations ≥10 ng/μl, corresponding to total yields ≥ 2 μg, were obtained for 94 % of the saliva specimens (*n* = 2344).

**Conclusions:**

In spite of inferior purity, the performance of saliva DNAs for microarray genotyping was excellent. Our results agree with other studies concluding that saliva collection is a viable alternative to blood. The potential to boost study enrollments and reduce subject discomfort is not necessarily offset by a reduction in genotyping efficiency. Saliva DNAs performed comparably to blood DNAs for PCR Sanger sequencing.

**Electronic supplementary material:**

The online version of this article (doi:10.1186/s12920-016-0172-y) contains supplementary material, which is available to authorized users.

## Background

Large epidemiological studies with thousands of participants are increasingly supplementing survey data with genomic DNA [1]. These studies require a simple, non-invasive method of sample collection that yields genomic DNA of adequate quality and quantity for high-throughput technologies [2]. Blood has traditionally been the primary source of genomic DNA, but saliva collection has recently emerged as a viable alternative [2, 3]. In addition to being less invasive, saliva collection has a lower overall cost, lower risk of infection, and simpler logistics. Stabilized saliva specimens can be stored at ambient temperatures for months, whereas blood must be frozen for long term storage, and protected from freeze/thaw cycles [2, 4]. Unlike blood collection, saliva collection does not require a trained phlebotomist; subjects need only to be provided simple directions, and may even donate saliva specimens by mail. These characteristics facilitate community outreach efforts by reducing costs for personnel training and effort, and eliminate the logistical complexities associated with transporting highly perishable blood specimens from remote locations. Saliva collection also leads to significantly higher response rates [1, 5], with one study finding a 72 % response rate for saliva collection versus 31 % for blood draws [1]. Another advantage is that saliva DNA has the potential to provide information about the oral microbiome. Despite these advantages, there is still reluctance among the scientific community to use saliva samples, which largely stems from concerns over reduced yield and quality of DNA [6–12].

Most studies agree that oral specimens yield lower quantities of DNA compared to blood [2, 4, 13, 14]. DNA isolated from saliva samples is often contaminated by foreign DNA from bacteria, fungi, and food remnants [2, 13, 15]. Non-human DNA content from saliva samples varies greatly among patients, with studies reporting non-human DNA yields ranging from 23 to 63 % of total DNA [16]. Despite concerns over low yield and variability among samples, previous studies have found that saliva collection still provides sufficient DNA for genotyping [1, 2, 4, 15, 17].

Research on the usability of saliva samples from older age groups remains limited, however. Subject age determines, in part, the number of epithelial cells found in saliva [18, 19]. A previous study found a strong positive correlation between subject age and DNA concentration from saliva samples, with children under age 12 having the lowest DNA concentration [18]. It is important to extend this research to older populations, as the elderly may have veins that are difficult to access for blood collection [2]. Additionally, saliva collection has been shown to reduce anxiety and increase participation rates in older participants [2]. Negative correlation between blood DNA yield and subject age was reported in a large prospective study by Caboux et al by assessing EPIC records of 50,000 subject DNA yields isolated from blood, 14 % of whom were ≥ 65 [20]. However, saliva collection in the elderly requires further investigation, as hyposalivation can interfere with specimen collection in this age group. Dry mouth has an incidence rate of 30 % in individuals over age 65 [21].

The Primary Open-Angle African-American Glaucoma Genetics (POAAGG) study cohort is the largest African-African primary-open angle glaucoma cohort recruited at a single institution (University of Pennsylvania, Department of Ophthalmology, Scheie Eye Institute) to date [22]. The growing size of the cohort (*n* = 5300), older age of POAAGG subjects, improvements in saliva stabilization technologies, and reductions in the amount of DNA needed for next-generation sequencing and genotyping applications have led us to consider saliva collection as the primary method for future subjects. The objective of this study is to examine how well DNA isolated from saliva samples performs, compared to DNA from blood, for array-based genotyping and sequencing. These results will inform the future method of DNA collection for the POAAGG study, as well as other large-scale studies requiring genomic DNA from older populations.

## Methods

### Subject recruitment and specimen collection

The POAAGG study is a five-year population-based project funded by the National Eye Institute of the National Institutes of Health. The study population consists of self-identified Blacks (African Americans, African descent, or African Caribbean). Although subjects as young as age 35 are potentially eligible, primary open-angle glaucoma is typically a disease of old age. Accordingly, enrollment efforts for controls have also preferentially targeted an older population, and the mean age of POAAAGG subjects is approximately 65. POAAGG subjects were recruited from the University of Pennsylvania from the Scheie Eye Institute, The Perelman Center for Advanced Medicine, and the Mercy Fitzgerald Hospital Ophthalmology satellite. All subjects provided informed written consent, in accordance with the tenets of the Declaration of Helsinki, under University of Pennsylvania IRB-approved protocol 815033.

Blood was collected by venipuncture in 10 ml purple top tubes with EDTA anticoagulant. The maximum volume of blood collected was 8 ml per tube, but sometimes less was obtained. These samples were frozen at −20° prior to DNA isolation. For saliva collection, subjects were asked to refrain from drinking or eating 30 min prior to donating specimens and to remove lipstick. Subjects experiencing dry mouth or difficulties with salivation were directed to massage their cheeks with a gentle circular motion to stimulate the salivary glands. Subjects struggling with hyposalivation were also offered packets of sugar, or a sugar substitute, and told to place a small amount on their tongues to induce salivation. A maximum volume of 2 ml of saliva per subject was collected in Oragene DISCOVER (OGR-500) self-collection kits (DNA Genotek, Canada), because we found that delivering more than one 2 ml saliva specimen in a single sitting was challenging for subjects, but most subjects were able to deliver this volume within a few minutes. The saliva specimens were mixed with stabilizing reagent within the collection tubes per manufacturer’s instructions, and these were stored at room temperature until DNA extraction.

### DNA extraction

DNA was isolated from freshly thawed blood samples using Gentra PureGene kits (Qiagen, Valencia, CA), and the optional RNase treatment step was included. DNA from saliva samples was extracted using the prepIT.L2P reagent (cat # PT-L2P-5, DNA Genotek, Canada) and precipitated with ethanol according to manufacturer’s instructions. The saliva DNA samples were RNAse treated by double digestion with RNase A and RNase T and re-precipitated using ethanol according to manufacturer’s instructions.

### DNA quantitation and sample selection

The concentrations of DNA from blood and saliva samples were determined using the fluorescence-based Quant iT dsDNA Board-Range (BR) assay kit (cat # Q-33130, Life Technologies, CA). Fluorescence was measured with a Tecan Infinite M 200 Pro multimode microplate reader (Tecan, NC). Two thousand nine hundred ten DNAs (566 from blood and 2344 from saliva) from the POAAGG cohort were used to evaluate DNA yields. During November 2014 the POAAGG study switched to from blood to saliva as the primary means of specimen collection, and all available saliva DNAs obtained since then were included. Blood DNAs which had been quantified using Nanodrop spectrophotometry were excluded from analysis to control for potential bias from different quantitation methods. DNA quantification by UV spectrometry may be confounded by RNA or other contamination, and may systematically overestimate DNA concentration. Accordingly, only the blood and saliva samples that had been quantified by the same automated fluorometry protocol were used to compare DNA yields. A subset of this group, 94 DNA samples (50 from blood and 44 from saliva), were selected for microarray analysis. These included 23 pairs of samples from which blood and saliva were obtained from the same individual. The selection of the group of 94 samples was deliberately weighted to include those having unusually high and low DNA concentrations, along with some saliva DNAs that were unusually turbid or discolored. UV absorption spectra from 220 to 340 nm and 260/280 and 260/230 absorbance ratios were also obtained for this group, using a Nanodrop ND-8000 spectrophotometer (Thermo Scientific, DE), and protein contamination was measured directly with the Qubit protein assay kit (cat # Q33211, Life Technologies, CA) with a Qubit 2.0 Fluorometer. The 1555 DNA samples chosen for sequencing comprised consecutive samples from early February 2013 to late October 2015, spanning the time interval during which the POAAGG study shifted from blood to saliva collection.

### Microarray genotyping and PCR Sanger sequencing

Ninety-four DNA samples were genotyped in two separate batches using the HumanOmniExpress 24v1 bead chip assay (Illumina, CA) on the Infinium platform by Illumina FastTrack Services (Illumina, San Diego, CA). The genotype calls were generated using the GenomeStudio genotyping module (GT). Cluster optimization, reproducibility analysis for paired samples, and data evaluation were also performed as per standard practices at Illumina FastTrack services. During cluster optimization 1822 markers were removed from 716,503 total markers on the array.

Saliva DNAs were used as templates for PCR targeting glaucoma-associated SNPs for two genes, *TMCO1* and *CDKN2B-AS1*. PCR was done in a reaction volume of 12 μl using Platinum Taq hot start DNA polymerase (#106566-034, ThermoFisher.com), dNTP mix (# 18427-088, ThermoFisher.com) and betaine (Sigma #B0300, http://www.sigmaaldrich.com). Each PCR reaction contained 1.2 μl of 10x Platinum Taq reaction buffer, 0.24 μl of 10 mM dNTPs, 0.96 μl of 50 mM MgCl_2_ (4 mM final), 2.4 μl of forward and reverse primer (2 pmol/μl), 3 μl saliva DNA, 0.096 μl Taq polymerase, 3.6 μl 5 M betaine, and 0.5 μl nuclease-free water. For *TMCO1*, 1555 DNA samples were tested, using forward primer ACCACAGGGAGCCTCTCGTT and reverse primer GCCCTGCCTGCTTTTTAGGGA. For *CDKN2B-AS1*, the same 1555 samples were tested, using forward primer GCGGAGAAGAATGTCCCGGC and reverse primer GCCAGGAAGGACGAGTCCCC. Thermal cycling was performed on an ABI 9700 instrument using a touchdown protocol: initial denaturation 95 deg C 5 min; 14 cycles: 94 deg 20 s, 63 deg to 56 deg with 0.5 deg decrement per cycle 20 s, 72 deg 45 s; 25 cycles: 94 deg 20 s, 56 deg 20 s, 72 deg 45 s; 72 deg 10 min; final hold at 10 deg. PCR products were cleaned up by digestion with shrimp alkaline phosphatase (SAP) and exonuclease 1 (Exo1). Cycle sequencing reactions were robotically assembled using a Biomek 3000 automated liquid handling system, with the BigDye Terminator v3.1 kit (#4337455, ThermoFisher.com). Cycle sequencing was done in a volume of 5 μl, containing 1.25 μl of a 4-fold or 8-fold dilution of the SAP/Exo1 digested PCR product, 1.25 μl sequencing primer (one of the PCR primers) at 5 pmol/μl, and 2.5 μl diluted BigDye v3.1 Ready Mix. Sequencing reactions were cleaned up with BigDye XTerminator kits (#4376485, ThermoFisher.com). Capillary electrophoresis was done on an ABI 3130xl genetic analyzer with 50 cm capillary array and POP-7 polymer (#4363785, ThemoFisher.com), and sequencing chromatograms were aligned, trimmed and scored using Sequencher 5.1 software (GeneCodes Corp, genecodes.com).

### Statistical analysis

Kernel density plots were created using the ggplot2 package in the R statistical package [23]. Comparisons between blood samples and saliva samples were made using t-tests for comparison of means and chi-squared tests for comparison of proportions. For the comparison of 23 paired samples with blood and saliva from the same subjects, a paired t-test was used. An F-test was used to test for equality of variance between two groups. All these statistical comparisons were made using SAS v9.3 (SAS Institute Inc., Cary, NC), and two-sided *p* < 0.05 was considered to be statistically significant. Sequencing results from blood vs. saliva DNAs were compared using a two-sided, two-sample proportion test with STATA v14.1 (StataCorp, College Station, TX).

## Results and discussion

### Quality and yield of DNA from saliva

**Fig. 1:**
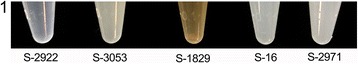
Examples of saliva DNAs having visible impurities. Sample S-2922 has a normal clear appearance, similar to DNAs extracted from blood. The others have various degrees of turbidity and/or brownish/reddish discoloration. Brownish saliva DNAs, S-3053 and S-1829, may have come from specimens contaminated by tobacco, food dyes or lipstick

The saliva samples from our predominately elderly study population yielded DNAs that were often highly viscous, with the majority having noticeable turbidity. Although subjects had been asked to not eat or drink 30 min prior to specimen donation, with the exception of placing a small amount of sugar or sugar substitute on the tongue to stimulate salivation when needed, visible contaminants such as food particles, lipstick, food coloring, tobacco, etc. were sometimes present in the saliva samples. In some cases, brownish discoloration, suspected to be tobacco-related, or reddish contamination (chewing gum, candy, or lipstick) carried through processing and were still visible in some of the purified DNA samples (Fig. 1).

Two thousand three hundred forty-four saliva specimens and 566 blood specimens were obtained, with both blood and saliva specimen obtained from 23 people for purposes of this study, and for a small number of individuals for whom the initial DNA extraction from blood was not successful. The demographics of the two groups of participants were very similar, with mean age 65 years and approximately 68 % female (Table 1). The mean (±SD) total yield of DNA from the 2344 saliva specimens was 35 ± 36 μg, as compared to the 106 ± 68 μg in the 566 blood specimens. However, after accounting for the smaller specimen collection volume that was attempted for saliva (2 ml) vs. blood (8 ml), the mean (±SD) yield of DNA per ml saliva specimen was 17.6 ± 17.8 μg/ml, which was significantly higher than that for blood specimens (13.2 ± 8.5 ug/ml, *p* < 0.0001). The higher yield of DNA per ml of saliva is necessarily offset by the presence of non-human DNA, which, as mentioned above, has been addressed by other studies. A failure rate of 6.0 % (141 subjects from 2344 total) was observed for saliva specimens, with failure defined as a final DNA concentration that was below 10 ng/μl of DNA, which corresponded to less than 2 μg yield in the minimum elution volume (200 μl). There was a weak negative correlation (Spearman correlation coefficient *r* = −0.1, *p* < 0.0001) between subject age and DNA yield from saliva samples whereas, the DNA yield from blood was not correlated with age (*r* = 0.04, *p* = 0.30).

**Table 1:** Demographic characteristics and DNA yields per specimen, corresponding to 2910 DNAs included in this study

	Blood (8 ml max)	Saliva (2 ml max)	*P*-value
No. samples	566	2344	
Age ± SD years	65 ± 12	65 ± 12	0.95
Female (%)	67.8	67.4	0.88
Mean ± SD for total DNA yield per specimen (μg)	106 ± 68	35 ± 36	*P* < 0.0001
Mean ± SD for DNA yield per ml of requested specimen (μg/ml)	13.2 ± 8.5	17.6 ± 17.8	*P* < 0.0001

Specimen collection volume was up to 8 ml for blood and up to 2 ml for saliva

**Fig. 2:**
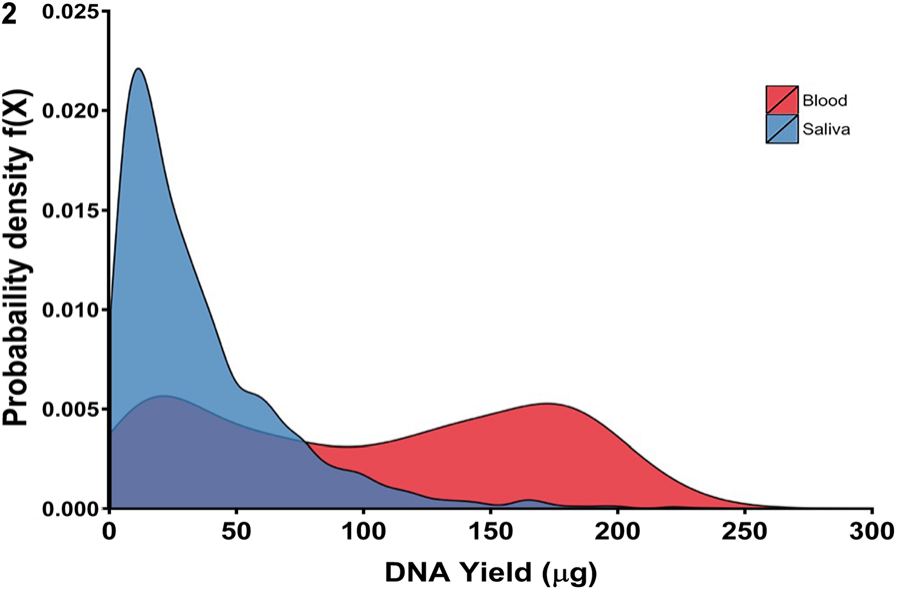
Density plot summarizing 2910 total DNA yields that were obtained from single blood (*n* = 566, 8 ml max) and saliva (*n* = 2344, 2 ml max) collection tubes

The total DNA yield distribution from the larger subset of the POAAGG cohort (566 blood DNAs and 2344 saliva DNAs) was evaluated by the Quant iT assay, and is illustrated as a kernel density plot in Fig. 2. The majority of saliva samples fall in the lower yield region of the plot, as expected, whereas the yield from blood tubes varies widely, with considerable overlap with the yield from saliva, in spite of the 4-fold larger maximum specimen volume (8 ml vs. 2 ml) that was attempted for blood. The distribution of DNA from saliva is relatively narrow with a single peak, whereas the blood DNA distribution is broad and bimodal. Although exact specimen collection volumes were not recorded, we believe this difference is because we often were unable to collect a full 8 ml blood specimen for a substantial fraction of our mostly elderly study population, whereas almost all subjects succeeded in supplying the 2 ml saliva specimen volume that was requested.

**Fig. 3:**
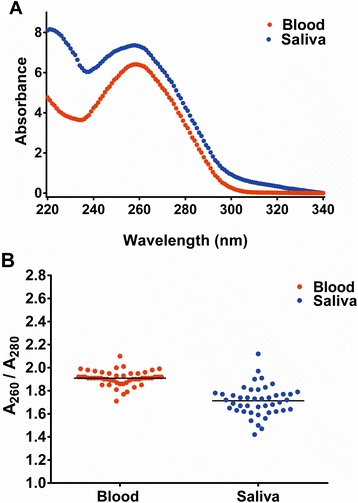
Comparisons of UV absorbance of blood vs. saliva DNAs. The average absorbance spectra of blood (*n* = 50) vs. saliva specimens (*n* = 44) are compared (**a**), and the distributions of 260:280 absorbance ratios around each mean, indicated by horizontal lines, are shown in (**b**)

Among the 94 samples that were selected for microarray genotyping, average UV absorbance for the 44 saliva DNA samples was higher than for the 50 blood DNA samples across the range from 230 nm to 340 nm (Fig. 3a). The higher absorbance at A_230_ nm may be due to the presence of relatively large amounts of carbohydrates and heavily glycosylated mucin in the saliva samples, or sugar that had been given to ameliorate dry mouth. The mean A_260/280_ ratio for saliva DNAs (1.71) was significantly lower than for blood (1.91) (*p* < .0001, Table 2, Fig. 3b). Furthermore, the A_260/280_ ratios for saliva DNAs were much more variable than for blood DNAs (*p* < 0.0001 for test of equal variance, Table 2, Fig. 3b). The 94 DNA concentrations and quality data, as measured by fluorescence and spectrophotometry are shown in Additional file 1: Table S1, and summarized in Table 2. It is important to note that the mean DNA concentration of the 44 saliva DNAs chosen for genotyping, 78.4 ng/μl, was more than 2-fold lower than for blood (175.3 ng/μl) (*p* < 0.0001, Table 2). A minimum concentration of 50 ng/μl is recommended for genotyping with the OmniExpress array, so this study deliberately included many saliva DNAs having sub-optimal concentrations for this purpose.

**Table 2:** Characteristics of the 94 blood and saliva DNA samples selected for genotyping

Sample source	Mean DNA conc (ng/μl)	Mean ± SD 260/280	Mean ± SD 260/230
Blood (*n* = 50)	175.3 ± 163	1.91 ± 0.06	1.50 ± 0.57
Saliva (*n* = 44)	78.4 ± 91.5	1.71 ± 0.13	1.00 ± 0.30
*P*-value	0.0006	*P* < 0.0001	*P* < 0.0001

The turbidity of many DNAs obtained from saliva (Fig. 1), together with the decreased 260/280 absorbance ratio for saliva DNA during spectrophotometry, prompted us to evaluate protein contamination in the 94 DNAs that had been selected for microarray genotyping. Using the Qubit Protein assay, the protein concentrations was below the level of detection in 42 (84 %) of blood samples and in 16 (36 %) of saliva samples (*P* < 0.0001, Additional file 1: Table S1). However, among those with protein above the detectable level, we observed no significant difference in the mean protein estimation from blood (116 ± 54 ng/μl) versus saliva DNA (119 ± 59 ng/μl).

### Genotyping efficiency, accuracy and PCR Sanger sequencing

**Fig. 4:**
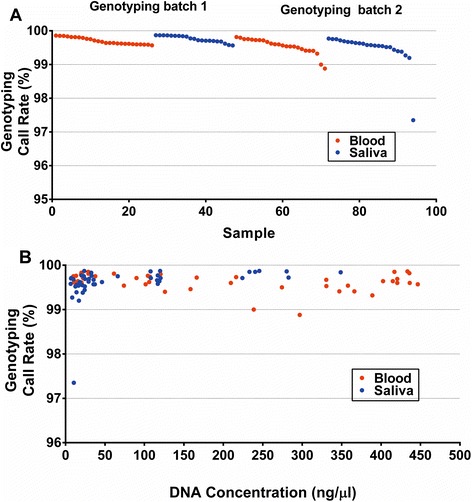
Comparison of genotyping call rates by Illumina Human OmniExpress microarrays. 94 DNAs from blood or saliva were genotyped in 2 batches (**a**), and call rates are plotted versus DNA concentration (**b**). The first batch of samples was deliberately enriched for DNAs having visible turbidity and/or abnormally low or high concentrations. The second batch contained 23 paired specimens: both blood and saliva collected from the same individuals

Surprisingly, the average genotyping call rates from the Illumina HumanOmniExpress 24v1 bead chip assay for blood and saliva DNAs were nearly identical: 99.62 vs 99.61 % from blood and saliva, respectively. The individual call rates for the 94 samples, along with age, gender, concentrations and comments on physical appearance of the DNA in solution after extraction are listed in Additional file 1: Table S1. The majority (59 %) of these subjects were age 65 or older. The genotyping call rates are plotted for the two-genotyping batches, with source tissues indicated, in Fig. 4a. If 98 % is considered the minimum threshold for success, then only one outlying sample, S-781 from saliva, failed; the paired blood DNA collected from the same individual yielded a typical passing call rate (99.5 %). With the exception of this one sample, all saliva DNAs yielded call rates > 99 %, whereas the 2^nd^ and 3^rd^ worst performing samples in this survey were blood DNAs (Fig. 4a). The genotype call rates were slightly lower in batch 2, due to optimized clustering for batch 1.

The relationship between genotyping call rate and DNA concentration of the samples is plotted in Fig. 4b. We observed genotyping call rates above 99 % on 92 of 94 samples, although the concentration of many was below 10 ng/μl, less than 20 % of the minimum (50 ng/μl) recommended for this assay. All low concentration blood DNAs (<25 ng/μl) yielded call rates 99 % or higher, whereas a low concentration (10.5 ng/μl) saliva DNA, S-781 had the lowest call rate (97 %) (Additional file 1: Table S1). The two worst performing blood samples, B-1898 and B-636 with call rates of 98.9 % and 99.0 %, had intermediate DNA concentrations 238 ng/μl and 296 ng/μl, suggesting no discernable effect of DNA concentration on the call rates for DNA from blood throughout the tested range of ~10 to ~400 ng/μl. Although all but one saliva DNA yielded call rates >99 %, the worst performing saliva samples were clustered at the lowest end of the concentration range (Fig. 4b). However, the average call rate (99.68 %) for blood DNAs, having concentrations ≤ 25 ng/μl, was only slightly higher than for dilute saliva DNAs only (99.45 %), and this difference is not significant (*p* = 0.08). For more concentrated DNAs, > 25 ng/μl, the average call rate for saliva DNAs (99.73 %) was actually higher than blood DNAs (99.6 %) (*p* = 0.003). In general, our results are consistent with those of Bahlo et al. [17] who concluded that genotyping with an Illumina platform was generally robust for saliva DNAs, even though these contain visible impurities and bacterial or other non-human DNA.

**Fig. 5:**
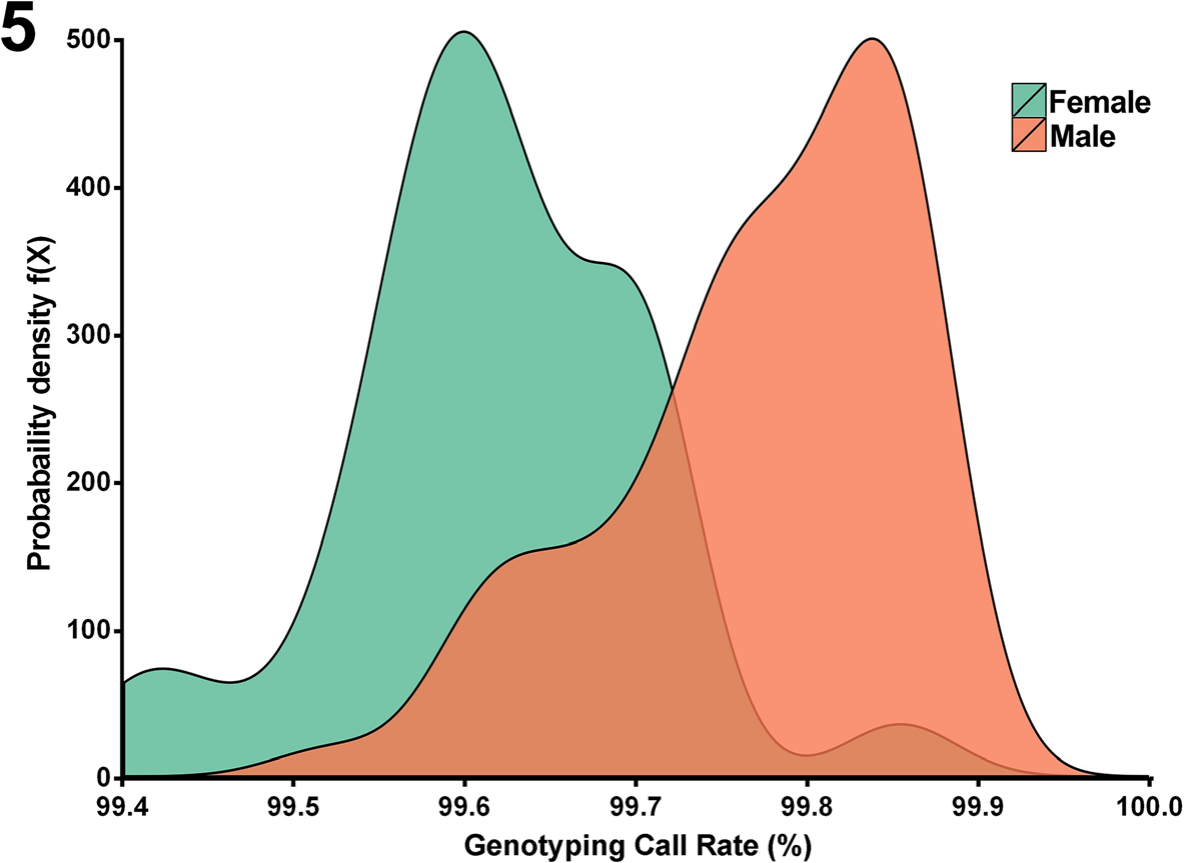
Comparison of genotyping call rates on the Illumina Human OmniExpress microarrays for male (*n* = 46) vs. female (*n* = 48) subjects

Genotyping call rates were expected to be slightly higher for males than females, due to the presence of Y chromosome markers on the array. The difference in the distributions between the genotyping call rates of males vs females is noticeable on the kernel density plot in Fig. 5, although this difference is small in absolute terms. The mean genotyping call rates were 99.5 % for females and 99.7 % for males.

Among 22 paired blood and saliva DNAs isolated from the same individuals, their call rate agreed very well, with a mean difference of 0.083 % (*p* = 0.46, paired t-test). One pair of samples was removed from this genotyping concordance estimate on account of the extreme outlier with low genotyping call rate of 97.35 %, saliva sample (S-781), discussed above.

One thousand five hundred fifty-five of the saliva DNAs were evaluated by PCR Sanger sequencing, targeting glaucoma-associated SNPs in or near the *CDKN2B-AS1* and *TMCO1* genes. High quality Sanger sequencing (Quality Value (QV) > 25, KB Basecaller) was obtained for ≥ 98.0 % of these samples for *CDKN2B-AS1* and *TMCO1* amplicons, from both blood (*n* = 297) and saliva (*n* = 1258) DNAs, and these success rates did not differ significantly (data not shown).

## Conclusion

Although DNAs extracted from saliva were inferior to those from blood in terms of physical appearance and standard measures of quality, their performance in array-based genotyping was excellent, and nearly indistinguishable from DNAs from blood. With the exception of a single saliva DNA sample (1 of 44) having a low call rate of 97.35 %, saliva-derived DNA samples yielded call rates > 99 %, with genotyping results that were highly concordant with blood DNA from the same subjects. The mean concordance of genotyping calls from the paired saliva-blood samples was at least 99.996 % (Additional file 1: Table S1). Saliva specimens yielded a minimum of 2 μg DNA at concentrations above 10 ng/μl for 94 % of specimens (*n* = 2344 extractions). High quality PCR Sanger sequencing data was obtained for ≥ 98 % of blood and saliva DNAs in two independent high throughput sequencing experiments (*n* = 1555 tested). Collection of saliva DNA has the potential to boost study enrollments, thereby increasing the statistical power of large population based studies such as the POAAGG project, while decreasing the personnel effort and training required to obtain DNA samples of adequate quality for microarray-based genotyping and sequencing.

## Additional file

Additional file 1: Table S1. Subject gender, age, DNA characteristics and genotyping results corresponding to the samples used for genotyping. (“B” sample IDs indicate DNA from blood and “S” sample IDs are DNA from saliva). (DOCX 31 kb)
