# Supplementary material for: Saliva DNA quality and genotyping efficiency in a predominantly elderly population
Source: BMC Med Genomics. 2016 Apr 7;9:17. doi: 10.1186/s12920-016-0172-y (PMC4823890; doi:10.1186/s12920-016-0172-y)
Supplement: Additional file 1: Table S1. — Subject gender, age, DNA characteristics and genotyping results corresponding to the samples used for genotyping. (“B” sample IDs indicate DNA from blood and “S” sample IDs are DNA from saliva). (DOCX 31 kb) [file 12920_2016_172_MOESM1_ESM.docx]

**Supplemental Table 1**. Subject gender, age, DNA characteristics and genotyping results corresponding to the samples used for genotyping. (“B” sample IDs indicate DNA from blood and “S” sample IDs are DNA from saliva)
